# Analysis of Electrome as a Tool for Plant Monitoring: Progress and Perspectives

**DOI:** 10.3390/plants14101500

**Published:** 2025-05-16

**Authors:** Elizaveta Kozlova, Lyubov Yudina, Ekaterina Sukhova, Vladimir Sukhov

**Affiliations:** Department of Biophysics, N.I. Lobachevsky State University of Nizhny Novgorod, 603950 Nizhny Novgorod, Russia; lyubovsurova@mail.ru (L.Y.); n.catherine@inbox.ru (E.S.); vssuh@mail.ru (V.S.)

**Keywords:** plant electrophysiology, plant electrical signals, electrome, mathematical modeling, plant stress

## Abstract

In recent years, the electromic approach, which is based on the ‘electrome’ concept, to the analysis of electrical activity in plants has become increasingly relevant, as it can allow the detection of early signs of stress and the classification of external factors on the basis of complex, systemic changes in electrical parameters. However, the mechanisms underlying the observed complex effects remain unresolved. This review describes the main electrical signals in plants and their influence on physiological processes and tolerance to abiotic stressors, discusses limitations of traditional methods of investigation of electrical activity, summarizes modern strategies for electrome analysis, and considers the prospect of applying mathematical modeling to interpret the electromic data. We suggest that the integration of the electromic approach and mathematical modeling can greatly enhance the ability to investigate plant electrical signaling, opening new ways for fundamental and applied research in plant electrophysiology.

## 1. Introduction

**Figure 1:**
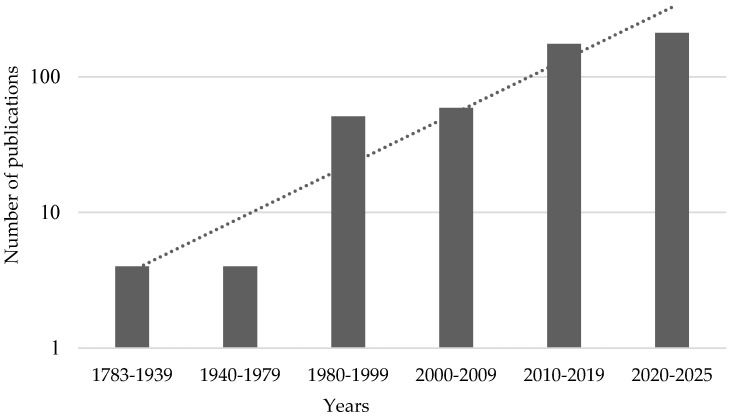
Dynamics of the number of publications devoted to investigation of plant electrical phenomena which are present in Dimensions database. Keyword “Plant electrical signals” was used for the search.

Electrical phenomena in plants were discovered in the 18th century, almost immediately after Galvani’s discovery of biological currents in animals [1]. The hypotheses about electrical activity in plants were outlined by Betholon in «De l’electricite des vegetaux» in 1783 [2], as well as by Burdon-Sanderson in 1873 [3] and Darwin in 1875 [4]. The earliest records of the electrical signals (ESs) in plants were registered by the Indian biophysicist J. C. Bose [5], who investigated electrical responses in motor plants such as Mimosa pudica. He hypothesized that the role of ESs in plants is similar to that in animals, and supposed that phloem vessels are the main way of the ES transmission in plants [1,5]. Since then, mechanisms and functions of electrical phenomena in plants are actively investigated [6]; the number of articles in this field is rapidly increasing, reaching over two hundred in the last five years (Figure 1).

Today, it is known that electrical signals strongly influence numerous physiological processes in higher plants and can increase their tolerance to action of stressors [7]; as a result, developing methods of analysis of characteristics of ESs is an important basic and applied task. Traditionally, such an analysis is based on ‘manual’ or ‘automatic’ revealing of electrical signals and further estimation of their characteristics [8].

However, alternative approach has been recently proposed to analyze plant electrical activity based on extracellular measurements [9,10,11]. In accordance with this approach, electrical signals and other types of changes in electrical activity can be complexly analyzed on the basis of concept of ‘electrome’, which is the totality of electrical phenomena in different scales of organization of plants. Based on this concept, revealing and identifying ESs (or other electrical responses) is not necessary to analyze changes in plant electrical activity under action of stressors; in contrast, these changes are detected on the basis of integral parameters of time-series of electrical potential records.

The complex analysis of electrical activity in plants is a powerful tool for detecting the action of stressors and revealing their types [12,13,14,15,16,17,18]. Considering basic and applied importance of developing methods for the complex analysis of plant electrical activity, our review summarizes the results of investigations in this field and discusses potential directions for future research.

## 2. Electrical Signals in Higher Plants: Brief Description

Traditionally, electrical phenomena in plants can be classified into steady-state potentials (e.g., resting potential) and local or propagating ESs, which are induced by a local action of stressors [19,20,21,22]. Depolarization electrical signals are well studied [22,23,24,25] and have been observed in many photosynthetic organisms, from charophyte algae to higher plants [26,27,28]. They include action potential (AP) [22,29] and variation potential (VP, sometimes called ‘slow-wave potential’). Action potential is considered to be a self-propagating ES with an impulse shape which can be induced by action of non-damaging stimuli; in contrast, VP is a response to action of extreme stressors (e.g., heating to 55–60 °C and further [30]), has a variable shape, and is based on propagation of non-electrical signals (chemical and/or hydraulic) [21,22]. Both AP and VP often have large amplitudes equaling to several tens of millivolts [7,10,20,22,31,32].

Besides the classical AP and VP, Zimmermann and co-workers [33,34] proposed a third type of electrical signals in plants: the system potential (SP), which is the hyperpolarization electrical signal. System potential also appears to be a propagating signal, but may have a smaller amplitude than AP and VP, generally not exceeding 30 mV [30,35,36,37]. The recent results show [35,36,37,38] that the hyperpolarization electrical signals can be induced by stressors with moderate intensities (e.g., moderate heating to 40 °C), which are widespread in natural conditions. System potential seems to be strongly related to VP [39]. Particularly, there is research [34,35] which shows that VP is observed near the zone of irritation; in contrast, SP may be observed with increasing distance from this zone. System potential can also be induced after the second application of the stressor [33]; notably, this stressor induces a VP during its first action. Additionally, the same stressor can induce both SP and VP during its action on different parts of the plant [30,39].

Molecular mechanisms of generation of ESs have been proposed for depolarization electrical signals [22,39]. Both VP and AP include depolarization and repolarization phases. In higher plants, the depolarization phase begins with an increase in the current of Ca^2+^ ions into the cell through Ca^2+^-channels of the plasma membrane; an increase in Ca^2+^ concentration causes reversible inactivation of H^+^-ATPase, leading to proton accumulation in the cytoplasm, and the activation of anion channels, leading to the release of Cl^−^ from the cell. Both processes lead to depolarization of the plasma membrane and consequent activation of outwardly rectifying K^+^-channels. The increase in K^+^ ion current into the apoplast forms the beginning of the repolarization phase. Further formation of repolarization is due to the closure of Ca^2+^ channels and a decrease in calcium ion concentration, which causes the reactivation of H^+^-ATPase and closure of anion channels. The difference in the shape and duration of VP and AP is probably due to the different dynamics of Ca^2+^ ion concentration in the cell, which is associated with the fact that potential-dependent Ca^2+^ channels are involved in the generation of AP, while ligand-dependent or mechanosensitive Ca^2+^ channels are involved in the generation of VP [22,39].

The mechanisms of generation of SP, a hyperpolarizing signal, remain understudied. The hypothesis of Zimmermann and co-workers [33] suggests that the main mechanism of SP generation is the activation of plasma membrane H^+^-ATPase. Potentially, such activation could be due to a moderate increase in the concentration of Ca^2+^ ions in the cytoplasm [35]; this assumption adequately explains the transformation of EP into SP with increasing distance from the stressor zone. However, such a hypothesis contradicts data on apoplast alkalinization during SP generation [33], which rather point to H^+^-ATPase inactivation. On the other hand, it is known that a moderate increase in cytosolic Ca^2+^ concentration can also induce inactivation of inwardly rectifying K^+^-channels and, hence, a slowing of K^+^ flux into the cell [22,39]. According to an alternative hypothesis of SP formation [39], simultaneous inactivation of H^+^-ATPase and inwardly rectifying K^+^-channels can cause both hyperpolarization (K^+^-channel inactivation predominates) and depolarization (H^+^-ATPase inactivation predominates); i.e., depending on the magnitude of Ca^2+^ flux into the cytoplasm, SP (moderate influx) or VP (large influx) can be observed in the plant.

The mechanisms of electrical signal propagation in plants remain incompletely investigated. It is known that AP propagation in higher plants is based on local electrical circuits in the symplast of parenchyma cells of vascular bundles and/or in phloem sieve elements [7,22,29]; i.e., AP is the self-propagating electrical signal. On the other hand, VP propagation is associated with the propagation of signals of non-electrical nature, which induce Ca^2+^ influx and electrical response [7,40]. The propagation of a hydraulic wave that activates mechanosensitive Ca^2+^-channels of the plasma membrane or a chemical agent (wound substance) that activates ligand-activated Ca^2+^-channels have been considered as such signals [19,22,23,40]; meanwhile, the hydraulic hypothesis seems to be in better alignment with ER propagation velocities. There are also hypotheses describing VP propagation based on the interaction between hydraulic and chemical signals [40,41]. The mechanism of SP propagation is currently debated [7,33]. Previously, we suggested that the common propagation mechanism of VP and SP is the passage of a hydraulic wave [39]; at the same time, a high amplitude of the hydraulic wave leads to the formation of a variable potential, and a low amplitude forms a system potential.

The main role of propagating electrical signals is thought to be to inform neighboring and distant cells of action of stressors to further induce a systemic adaptive response to adverse factors, which often involves changes in numerous physiological processes [7,20,22]. Particularly, ESs induce the expression of defense genes [42,43], production of stress phytohormones [25,44], decrease of photosynthesis [30,36,38,45], suppression of phloem mass-flow [46,47], stimulation of respiration [48], changes in transpiration [49], etc. ES-induced increasing plant tolerance to action of stressors (including stimulation of photosynthetic tolerance) has also been shown in some studies [38,50,51,52,53,54,55]; this increase is related to photosynthetic and transpiration changes and, probably, to other physiological responses [7]. The increased tolerance to action of abiotic stressors can be induced by all electrical signals, including AP [50,51], VP [53,55], and SP [38]. It should be noted that environmental conditions, including drought [38] and chronic radiation exposure [55], modify the influence of ESs on plant tolerance; potentially, this effect can be related to changes in photosynthetic responses induced by electrical signals [36,38].

The mechanisms of influence of electrical signals on physiological processes are not fully clear [7]. It is likely that one of the key mechanisms linking electrical signals to subsequent physiological responses may be a Ca^2+^-dependent change in the absolute activity of H^+^-ATPase that develops during the generation of AP, VP, and SP in higher plants [7,39]. In particular, it has been shown that the inactivation of H^+^-ATPase leads to inhibition of photosynthetic processes [56]; apparently, such a response can be mediated by an increase in the pH of the apoplast and a decrease in the pH of the cytoplasm, stroma, and lumen of chloroplasts [39,57]. It is possible that changes in H^+^-ATPase activity can participate in the influence of ESs on other physiological responses, e.g., on production of jasmonic acid [44] or transpiration [53].

Thus, ESs, which are induced by the local action of stressors, are likely to play an important role in plant life, influencing numerous physiological processes and participating in adaptation of plants to environmental changes. It means that the measurement and analysis of these signals can be potentially used for agricultural and ecological monitoring; particularly, revealing the induction of ESs in ‘reference’ plants or changes in characteristics of these signals can be informative. In addition, it can be potentially used for the estimation of changes in biosensors based on plants.

## 3. Limitations of Traditional Investigations of Electrical Signals and Plant ‘Electrome’ Concept

Traditionally, the ‘manual’ identification of ESs (or other electrical responses) and further analysis of their parameters are used in investigation of electrical signaling in plants. There are few studies (e.g., [8]) that describe the ‘automatic’ identification of ESs, based on the wavelet analysis. It should be noted that both ‘manual’ and ‘automatic’ analysis of ESs have similar principles: an individual electrical signal should be revealed and characterized. However, classical studies on plant electrophysiology, based on the identification of specific electrical responses and the estimation of their types and parameters, often encounter a number of problems.

Firstly, the mechanisms of electrical responses occurring in the stressed zone are extremely diverse and may depend on the type of stressor [58], which complicates the form of such responses and makes their study difficult. The characteristics of electrical signals induced by external stimuli can also be influenced by the individual physiological status of the plant [59], growing conditions [60,61], and long-term exposure to damaging factors [37,38,55]. The laboratory conditions in which studies typically take place may differ from natural conditions, leading to modification of electrical activity [62]. In addition, electrical responses can be induced by low or moderate intensity stimuli [62,63], which are difficult to control in natural conditions.

Secondly, electrical signals can have low amplitudes (10–25 mV or less) and/or complex shapes that are not amenable to standard analysis [35,36,37,38,64,65,66], making them difficult to detect and parameterize. Meanwhile, low-amplitude signals affect plant physiological processes, including photosynthesis [36,37,38,64], and are involved in plant adaptation to stressors, including biotic injury [67] and drought [38]; i.e., such ESs also play an important role in plant’s life. The involvement of low-amplitude electrical signals in physiological regulation is further supported by the detection of significant changes in the concentrations of H^+^, Cl^−^, and, to a lesser extent, Ca^2+^ in the apoplast (substomatal cavities) during the development of such signals [68], as changes in ion concentrations are a likely mechanism for the induction of plant physiological responses during the propagation of ESs [7].

Thirdly, another factor complicating the classical analysis of electrical activity is the generation of AP-like spikes (also having a small amplitude), which in some cases develop against the background of a long depolarization wave during VP propagation, which is a key electrical signal with high amplitude in terrestrial plants. The parameters of AP-like spikes depend on the distance to the lesion zone, stressor properties, and individual plant status, forming irregular shape of variation potentials, thus complicating the analysis of electrical signal parameters; at the same time, the possible specific role of such spikes in the development of the physiological response to stressors is still not clear [39,40]. Significant shape variability is also inherent in the SP [33,34] and electrical signals with low amplitudes [38]. The latter can be depolarizing, hyperpolarizing, and multiphasic signals [35,37,38], and the types of signals can also change with increasing distance from the stressor zone. In the case of AP, the probability of its forming in response to stressors depends on environmental conditions [7,39]. Particularly, the propagation of AP in higher plants is mainly observed in stable and favorable conditions [7].

Fourthly, for stressor-induced ESs, there is the phenomenon of mixed electrical potential waves, representing the superposition of AP and VP, or the presence of AP-like spikes on the VP background [23,69,70], probably mediated by Ca^2+^ ions flux. These complex signals require additional analysis of their shape.

Finally, the very principle of recording electrical potential may influence the data obtained: non-invasive superficial methods may be limited by the cuticular shielding of the potential, while invasive methods may be limited by the complexity of the technique or the need for long adaptation to compensate for damage to the tissue in which the electrode is inserted [71].

It should be noted that these reasons, which limit traditional analysis of the propagating electrical signals, are also important for local ESs (local electrical responses), which are formed in the zone of direct action of stressors; however, these responses have additional reasons of changeability which are related to specific changes in ion transport across the plasma membrane under the action of specific stressors [72,73,74].

In addition to noted points related to electrical signals, the efficiency of traditional analysis of electrical activity can be disrupted through induction of periodic oscillations and stochastic electrical fluctuations [61], which can be formed without direct action of stimuli; these electrical responses are insufficiently studied now. The presence of random activity in plant tissues was first noted by Bose [1]; the study of oscillations in biological systems developed in the second half of the 20th century [75,76,77]. It has been hypothesized that oscillations are related to the operation of the vesicular system of cells, and local osmotic regulation process; presumably, they may also be involved in the dissemination of information across different plant tissues [78]. Among the main processes in which rhythmic changes in ion flux dynamics are likely to be involved are growth (in the pollen tube or shoot apex), transport in the root system, immune response formation, and symbiotic relationships (mycorrhiza or nodulation) [79,80,81]. In addition, oscillations may occur in response to rhythmic changes in lighting [82,83]. Also, there are several studies that demonstrate spontaneous fluctuations of electrical potential in plant tissues, with a number of studies showing a change in the nature of fluctuations in response to external stimuli, such as changes in light intensity or temperature, or to variation in the time of day [62,63,76,84].

**Figure 2:**
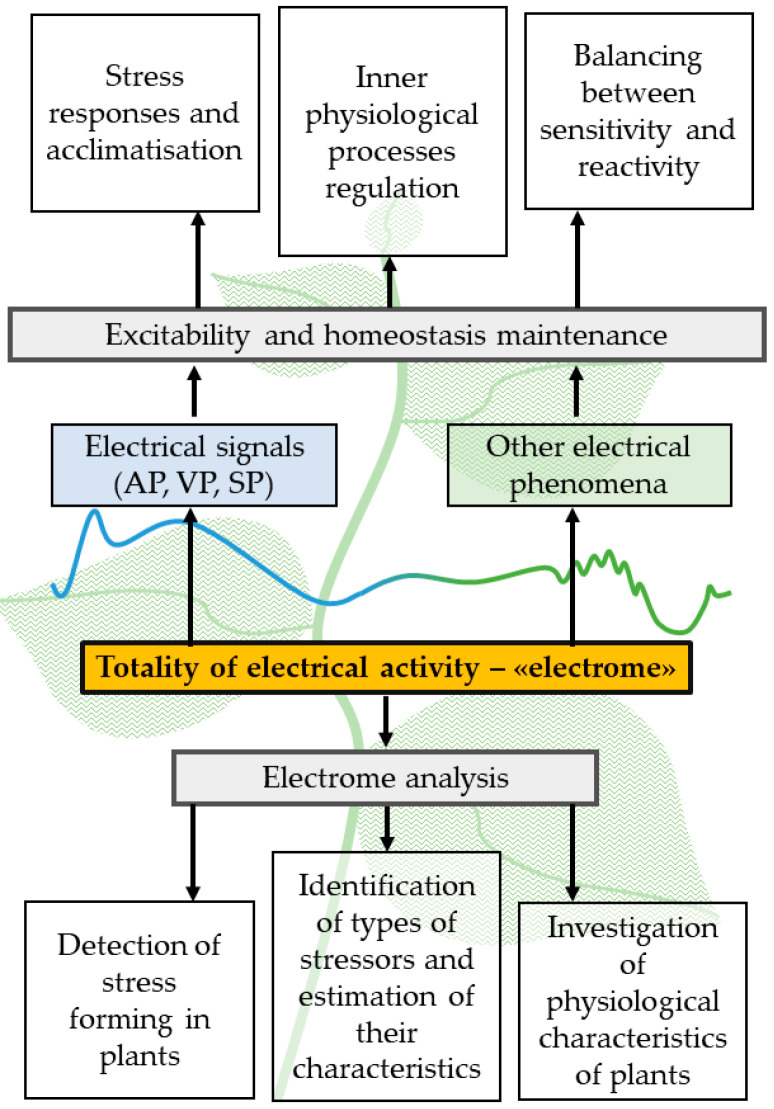
A proposed scheme for the function of the plant electrome as a totality of electrical phenomena in plants. The plant electrome, which can show signs of the presence of a self-organized critical state, may act as an important mediator of plant response to stimuli (external and internal) and the maintenance of homeostasis, being in balance between reactivity and sensitivity.

Thus, the electrophysiological responses of plant organisms are highly diverse, which may be related to an inability to avoid threats due to sessile lifestyle [85]. The totality of these responses, including slow shifts in resting potential magnitude, local electrical responses, oscillations, and fluctuations, ‘classical’ stress ESs (AP, VP, and SP), and low-amplitude electrical signals, can be unified by the concept of plant electrome, which is ‘the totality of the ionic dynamics in different scales of plant organization, engendering a constant electrical activity’ [9,10,11]. The concept of the electrome opens the possibility for a comprehensive analysis of plant electrical activity, which can be carried out without identifying specific electrical signals and analyzing their parameters. It may also help to describe plant’s adaptive strategies, for example, balance between resilience and reactivity or resonant response occurrence [7] (Figure 2).

Recent research has increasingly revealed the role of the electrome for plant function, growth, and development, as well as their interaction with the environment and adaptability [11,86]. Above all, a number of studies by Souza and co-workers have shown that complex changes in electrical activity (electrome changes) can be observed when plants are exposed to a wide range of abiotic stressors, including osmotic stress [9,15], low light [9], and localized effects of mechanical damage, heat shock, or a combination of both [87], and the changes detected depend on the type of stressor acting. It is important that specific sensitivity of total electrical activity of plants to the action of different chemical agents [13,14,88] and to light pulses with different durations [12] have also been shown by Chatterjee and co-workers. The last group of results supports the efficiency of complex analysis of total electrical activity (i.e., analysis of electrome) to detect the action of stressors on plants.

## 4. Methods of Measuring Electrical Activity and Approaches to Analyzing Electrome Parameters in Plants

The concept of the electrome implies a comprehensive study of plant electrical activity, which involves both recording such activity in plants and subsequent analysis of the obtained data. The most effective methods for measuring plant electrical activity are microelectrode measurements of electrical potential differences at the plasma membrane, including registration of phloem cell electrical potentials via aphid styles, voltage and patch clamp, etc. [22,29,89,90]. Despite the high accuracy of microelectrode measurements [29], they have several limitations: (i) the measurements are invasive and can lead to cell damage, which increases the difficulty of using the method and limits the length of recordings, (ii) they require expensive equipment and can only be performed in laboratory conditions, (iii) simultaneous measurements can be performed in a limited number of cells (often in a single cell) [22,71,91]. Such specific characteristics of microelectrode measurements allow them to be widely used to study the mechanisms of electrical responses of individual cells [22,92,93,94] but limit their effectiveness for the study of electrome parameters, as such measurements reflect little of the complex electrical activity in plants.

Within the macroelectrode technique, measuring electrodes can contact the plant object both invasively (by inserting them into the plant tissue [87,95]) or non-invasively (by connecting them to the plant surface through ionic contact [55,62,96,97], agar [45] or conductive gel [35]). Often, macroelectrode measurements allow the realization of multichannel analysis [35,36]; herewith, each macroelectrode contacts a separate group of excitable cells, reflecting their complex electrical response [59]. Such features of macroelectrode measurements reduce their effectiveness for accurate estimation of membrane potential, while making them a promising tool for comprehensive analysis of plant electrical activity [8,12,13,14,59,87], including the study of electrome parameters. An additional advantage of the macroelectrode technique is the potential for its use in open-air conditions.

Among measurement techniques, microelectrode and microneedle arrays are developing. It is supposed to overcome the limitation of both invasive microelectrode (the need for long-term adaptation, applicability only in laboratory conditions, and a limited number of registration channels) and macroelectrode techniques (cuticular electrical shielding). Such systems provide simultaneous recordings of electrical activity in several areas located at rather small distances from each other, thus allowing the analysis of, among other things, the electrical connections of plant cells [71,98,99,100].

Analyzing the obtained data is the next key step in the study of electrical activity in plants. According to the traditional approach, such analysis may rely on identifying electrical responses of a specific type in a recording and then determining the parameters of such responses, including threshold magnitude, amplitude, duration, propagation velocity, and others (see, e.g., [33,64,101,102]). In many studies, the identification of electrical signal parameters is performed based on the researcher’s experience; however, there are studies [8,103,104] in which the task of identifying electrical response parameters is implemented automatically using wavelet transform (WT). The WT method is based on the use of a function that imitates the basic form of the target response; when performing the WT, it searches for corresponding elements of the recording and estimates the parameters of the found elements in the time series (first of all onset and duration) [8,105,106]. It should be noted that WT allows estimation of parameters of specific electrical responses (including the time of initiation and duration of these responses) in a noisy recording; however, this method does not allow a comprehensive assessment of the characteristics of electrical activity (i.e., in its basic form, it is not applicable to electrome studies).

The analysis of the plant electrome requires more complex processing of experimental data that may allow us to identify not only typical electrical responses, but also changes in the electrical potential of the organism under study as a whole. As already mentioned, data for such analysis can be obtained using macroelectrode measurements; when recorded over long periods of time, such data represent typical time series suitable for further processing [8,10,88,107]. The acquisition of electrome data, sometimes referred to as electrophytography (EPG) [70,78,108], may require a certain approach to the design of the experiment, ensuring a long, multi-hour adaptation of the plant [18,109,110], normalization and monitoring of environmental parameters, and parallel recording of several physiological parameters other than bioelectrical potentials.

At the same time, time series of surface potential difference values obtained by a pair of macroelectrodes placed side by side on a plant plot are often used for analysis (i.e., single-channel measurement is used) [13,95].

Firstly, measured time series can be analyzed using basic methods of descriptive statistics, which reflect the general picture of changes and set the basis for further analysis. Such methods include the calculation of the standard deviation of the measured value, its minima and maxima, mean value, kurtosis, asymmetry, and dispersion [66,110,111,112]. These characteristics may describe the most general characteristics of the surface potential magnitude distribution determined from its time series.

Then, a classical EEG-like signal analysis may be performed. For time series of surface potential values, (i) linear correlation [17,70] is calculated, showing the bond between the analyzed data series, (ii) autocorrelation function [17,110,111], showing possible periodicity of the recorded surface potential dynamics, (iii) approximate entropy (ApEn) [15,17,70,87,109,110,113] or, alternatively, (iv) Lempel–Ziv complexity [97,114], showing the randomness of the time series of electric potential values, and (v) detrended fluctuation analysis (DTA) [87], showing the fractality of the measured values. From such series, frequency spectra of the potential value distribution are obtained using fast Fourier transform (FFT), and based on them (i) power spectral density (PSD, reflects how the average power of the waves is distributed through the frequencies or measures the energy variation in a signal distributed over the measured frequencies) [15,66,97,109,111,113], (ii) probability density function of the noise (PDF, demonstrates the probability of a variable to occur at a certain point of the histogram through a regression of the distribution values) [15,70,111], and (iii) average band power (ABP, analyzes specific frequencies of the signal) [87]. The calculation of β-exponents from PSD can indicate a type of noise that can vary with plant condition [66].

The mentioned parameters of surface potential dynamics can be used for electrome analysis and, as a corollary, for the study of general features of electrical activity processes in plants (the assessment of electrical connectivity of individual plant areas, detection of periodic patterns in electrical activity, determination of chaos and fractality in such activity, detection of self-organization effects, etc.). However, from a practical point of view, an important task of electrome analysis is to assess the state of the plant (and the fact that stressors of different nature are acting on it) based on measurements of electrical potential [88]. In this regard, an important method of analyzing bioelectrical processes in plants is machine learning [86,107], which allows solving the problem of classifying plants by their condition (or by the type of stressors acting on them) based on the analysis of time series of their electrical activity. It was shown that analysis of electrical activity may be based on different types of classifiers, like Bayesian, k-Nearest neighbor, Adaboost, support vector machine, ensemble boosted trees, gradient boosted trees, and others [14,17,88,95,107,111,113]. Examples of successful applications of machine learning analysis include the differentiation of fruit ripening stages [113], detection of dormancy states and effects on plants and stimuli [18], infestation by spider mites [95], and features of plant water balance and detection of drought and/or salinity stress [71,107,115] based on the analysis of electrome characteristics. In addition, several studies [14,88] have demonstrated the ability to classify and segregate plant electrical responses based on the type of chemical stressor. The analysis of plant electrical activity recordings using deep learning algorithms is also being developed [61,66,83]. High prediction accuracy in a number of studies—above 90% for machine [14,71] and deep learning [61,66]—emphasize the promising development of this direction for the analysis of electrical activity.

Overall, it can be noted that there is now a well-developed approach to analyzing plant condition based on complex electrome analysis (using surface potential time series). Further development of protocols for analyzing plant electrome, including machine and deep learning, will allow their application in environmental monitoring and agriculture, determining the cause of plant stress [13,14,88].

## 5. Perspectives of Using Mathematical Models for Improving the Electrome Analysis

As mentioned above, electrome analysis may be a fairly effective tool for the general assessment of plant state, including the identification of stressors. However, the use of complex electrical activity analysis methods to investigate specific mechanisms of plant responses to environmental factors is limited by the complexity of the biological system, which simultaneously includes a significant number of different types of interacting cells with different forms of electrical activity [30,116]; individual variability of parameters of such cells (e.g., their plasma membrane H^+^-ATPase activity [117]) is also highly expected. As a consequence, it can be anticipated that the results of standard methods for investigating the mechanisms of electrical responses in plants, including inhibitor analysis [45] or measurements of intra- and extracellular ionic concentrations [116], may be considered as very challenging to interpret.

An alternative approach to the interpretation of the results of complex analysis of electrical activity and electrome in plants may be based on the development of mathematical models of electrogenesis of plant cell ensembles. In recent decades, several mathematical models describing electrical activity at the cell level have been developed in the field of plant biophysics [118]. More specifically, a number of models for the generation of action potential have been developed based on a detailed description of ion fluxes [119,120,121,122,123,124]. Such models rely on various combinations of descriptions of the main mechanisms of electrogenesis: ionic channels, Ca^2+^-ATPase, H^+^-ATPase, secondary active transporters on the plasmalemma and, in some studies, on the tonoplast; the regulatory influence of Ca^2+^ on ion transport systems has also been taken into account. Similar approaches relying on the description of plant cell electrical activity can be also used for modeling VP [125], local electrical signals (local electrical responses) [117,120], stochastic electrical fluctuations [126], and rhythmic oscillations of membrane potential in cells or cell groups of higher plants [79,127,128,129,130,131].

It is worth noting that models of electrical responses in some cases rely on the description of an ensemble of excitable cells with local electrical coupling [118]. On the one hand, such an approach allows us to investigate the role of cooperation of different cells or stochastic heterogeneity of their parameters in the formation of electrical responses [117,118]; on the other hand, such models are used to describe the active propagation of electrical signals across the symplast of excitable cells [132,133]. Models describing ensembles of interacting cells have considerable potential to explain the interaction of noisy networks. A number of studies have demonstrated that heterogeneity of the initial activity of H^+^-ATPases can lead to a shift in the threshold of generation of electrical signals and induce the stochastic fluctuations of membrane potential [117,126].

Another approach to describing the propagation of electrical signals in plants may be the one to describe the propagation of non-electrical signals inducing local electrical responses. Thus, for VP propagation, models describing its induction by the propagation of wound substance [125,134,135] or hydraulic wave [136] have been proposed. In particular, VP models may rely on a description of the active propagation of wound substance. Thus, Evans and colleagues [134] proposed a mathematical model describing ROS-dependent propagation of VP in an array of interconnected cells and its integration with Ca^2+^-wave propagation; secondary ROS production was taken into account. Such a model is consistent with one of the previously proposed hypotheses on the mechanism of VP propagation [19]. Notably, there are studies [137,138,139] in which chemical signal transduction is also used to model information transfer by action potentials in higher plants; this takes into account, in particular, the functioning of ensembles of excitable cells with serial or parallel connections.

In addition, mathematical models of electrical activity can be related to descriptions of physiological processes [118]. Particularly, there are models describing relationships of electrogenesis to photosynthetic processes [118,140], stomata opening [141,142,143,144], or phytohormone production [144]. These models can potentially be used to describe physiological responses induced by electrical signals; however, there are only a few studies that analyze this problem (see our review [39]).

When considering the reliability of the data provided by modeling, it is common to compare it with data from an experiment. A number of papers [120,124,134] demonstrate the high relevance of mathematical prediction, which is another argument for the usefulness of modeling as a tool.

Thus, to the present day, there is a rather extensive base of detailed mathematical models for plant electrogenesis. Such models not only describe the basic mechanisms of generation of electrical signals and other electrical responses at the level of individual cells, but also can take into account electrical, chemical, and hydraulic interactions between individual cells, thus describing the sum of electrical activity of large areas of the plant. In particular, some developed models allow describing the results of macroelectrode registration as the sum of electrical responses of individual cells of a plant section [117,126]; at the same time, the shape of the integrated electrical signal may differ significantly from the individual signal under conditions of low electrical coupling between cells [126]. On the other hand, the developed models have the potential to investigate the influence of electrical signals on physiological processes [39,118].

Considering these points, the models of plant electrical activity are an effective tool to analyze complex mechanisms of electrical responses under the action of various stressors, including theoretical investigations of roles of specific molecular transporters, interactions between cells and parts of plant and, perhaps, physiological processes in plants [118]. It particularly means that the models can be used to increase efficiency of complex investigations of plant electrome. There are the following potential ways of this increasing. (i) Revealing mechanisms and improving interpretation of changes in electrome parameters using analysis of the mathematical models of plant electrical activity. Particularly, the analysis of the models can show relationships between changes in specific parameters of electrome and changes in specific electrophysiological characteristics of plants (e.g., activity of ion transporters, values of electrical conductance between cells, heterogeneity of these values, parameters of hydraulic and chemical signals in plant, etc.). Models may also describe synchronization of cell ensembles and, therefore, the occurrence of a self-organized critical state [7]; further analysis of modeled data, performed using electrome analysis methods (as described above), may show dependence of its emergent parameters, such as fractality [145] or functional coupling, on model’s specific parameters. (ii) Model-based prediction of parameters of electrome which will be sensitive to action of specific stressors can improve efficiency of complex analysis of electrical activity of plants. (iii) Model-based revealing optimal methodological parameters for measurements of electrical activity in plants. Potentially, it can theoretically show optimal positions of electrodes, their number, distances between them, the duration and frequency of measurements, and other parameters. (iv) Clarification relationships between parameters of electrome and physiological characteristics of plants including their physiological responses on action of stressors. It can be based on both more effective interpretations of known electrome parameters and revealing new parameters, which are sensitive to physiological processes.

Thus, despite potential limitations (e.g., the inability to completely describe a system with an excess of factors, including variable external conditions), it can be expected that the integration of mathematical models of electrical activity in plants, designed to simulate electrome time series dynamics (especially, models based on the description of ensembles of excitable cells with local electrical coupling [117,126]) may be an effective tool for improving plant investigations on the basis of the electrome concept.

**Figure 3:**
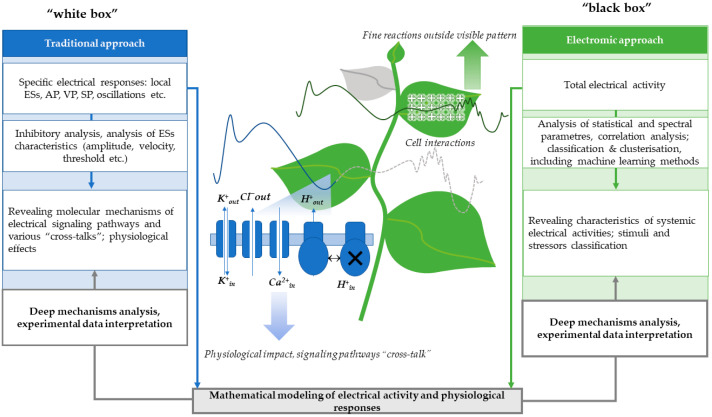
Proposed scheme for improving traditional and electromic approaches in plant electrophysiology. The traditional approach to plant electrical activity analysis can be resembled to a ‘white box’ model, where known mechanisms underlie specific electrical events, including local electrical signals (ESs), action potential (AP), variation potential (VP), system potential (SP), oscillations, and others. On the other hand, the electromic approach can be conceptualized as a ‘black box’, capturing emergent effects of collective electrical activity without fully deciphering the underlying processes. However, by applying mathematical modeling to both approaches, improving data interpretation and enhancing analytical capabilities may be achieved.

In summary, we illustrate a potential way of improving both traditional and electromic approaches in studying plant electrical activity in Figure 3.

## 6. Conclusions

Electrical activity in plants, including electrical signals, is an important mechanism of their adaptive response to the action of environmental stressors. The complex analysis, based on the electrome concept, is an effective tool to detect action of specific stressors on the plant and to estimate changes in general characteristics of its electrogenesis, which is important in the development of high-efficiency farming [146,147]. However, the efficiency of the electrome analysis for revealing specific mechanisms of changes in plant electrical activity and physiological responses is limited. The development of mathematical models of electrical responses in plants, including models of electrogenesis in individual cells, models of interactions between these cells, and models of physiological responses, is a perspective tool for increasing the efficiency of the complex analysis of the plant electrical activity on the basis of the electrome concept.

## Abbreviations

The following abbreviations are used in this manuscript:

AP	Action potential
EEG	Electroencephalography
EPG	Electrophytography
ESs	Electrical signals
ROS	Reactive oxygen species
SP	System potential
VP	Variation potential
WT	Wavelet transform
